# Evidence of *Yersinia pestis *DNA from fleas in an endemic plague area of Zambia

**DOI:** 10.1186/1756-0500-5-72

**Published:** 2012-01-26

**Authors:** Bernard M Hang'ombe, Ichiro Nakamura, Kenny L Samui, Davy Kaile, Aaron S Mweene, Bukheti S Kilonzo, Hirofumi Sawa, Chihiro Sugimoto, Brendan W Wren

**Affiliations:** 1 School of Veterinary Medicine, University of Zambia, Lusaka, Zambia; 2 Hokkaido University Research Center for Zoonosis Control, Sapporo, Japan; 3 Namwala District Medical officer, Namwala District, Zambia; 4 Sokoine University of Agriculture, Sokoine, Tanzania; 5 Department of Pathogen Biology, London School of Hygiene & Tropical Medicine, London, UK

## Abstract

**Background:**

*Yersinia pestis *is a bacterium that causes plague which infects a variety of mammals throughout the world. The disease is usually transmitted among wild rodents through a flea vector. The sources and routes of transmission of plague are poorly researched in Africa, yet remains a concern in several sub-Saharan countries. In Zambia, the disease has been reported on annual basis with up to 20 cases per year, without investigating animal reservoirs or vectors that may be responsible in the maintenance and propagation of the bacterium. In this study, we undertook plague surveillance by using PCR amplification of the plasminogen activator gene in fleas.

**Findings:**

*Xenopsylla *species of fleas were collected from 83 rodents trapped in a plague endemic area of Zambia. Of these rodents 5 had fleas positive (6.02%) for *Y. pestis *plasminogen activator gene. All the *Y. pestis *positive rodents were gerbils.

**Conclusions:**

We conclude that fleas may be responsible in the transmission of *Y. pestis *and that PCR may provide means of plague surveillance in the endemic areas of Zambia.

## Background

*Yersinia pestis *is a Gram-negative bacterium that causes plague, which primarily infects small mammals and is cycled from infected to uninfected hosts by fleas [1]. The disease persists in many parts of the world with 90% of the plague cases being reported to the World Health Organization each year, come from Africa where public health and living conditions are poor [2,3]. The natural foci of plague are spread worldwide, mainly in the rodent and flea vector reservoirs. In Zambia, cases of plague have occurred in the eastern and southern parts of the country as periodic epizootics [4-6]. The first confirmed major outbreak of plague in Zambia was in the Southern Province, Namwala district in December 1996-February 1997, where 267 human cases were reported, out of which 26 people died [5]. Since then, sporadic outbreaks occur in this area of Zambia.

The evidence of plague infection with *Y. pestis *is usually observed in several rodent and flea species [1,7,8]. Some rodent species may become bacteraemic upon exposure to *Y. pestis *and therefore serve as sources of infectious bloodmeals for fleas that transmit the pathogen [9]. The oriental rat flea *Xenopsylla cheopis *and the human flea *Pulex irritans *are thought to be important arthropod vectors in transmitting plague to humans [10]. *X. cheopis *is an efficient vector because of its proventriculus, which creates a location for growth of *Y. pestis*. The flea becomes blocked with *Y. pestis *and then it is unable to swallow a full blood meal [8,11,12]. In an attempt by the flea to dislodge the blockage, the flea infects new mammalian hosts. There are other flea species implicated as primary vectors but these may clear *Y. pestis *more quickly, such that only excreta of the flea or the crushing of its body may infect the host in contact with the arthropod [13-15]. Our study is the first attempt, to investigate the involvement of fleas as plague reservoirs or vectors in Zambia. Fleas of *Xenopsylla *and *Ctenocephalides *species have been observed to be abundant at times of plague epizootics in Zambia [5]. In this study, we used PCR to rapidly identify *Y. pestis *infected fleas in a plague endemic focus by detecting the plasmid encoded plasminogen activator gene [1]. The determination of the prevalence and distribution of *Y. pestis *in fleas has been suggested to be an important part of plague surveillance [1,16] in areas where disease outbreaks are common in the human population.

## Methods

**Figure 1:**
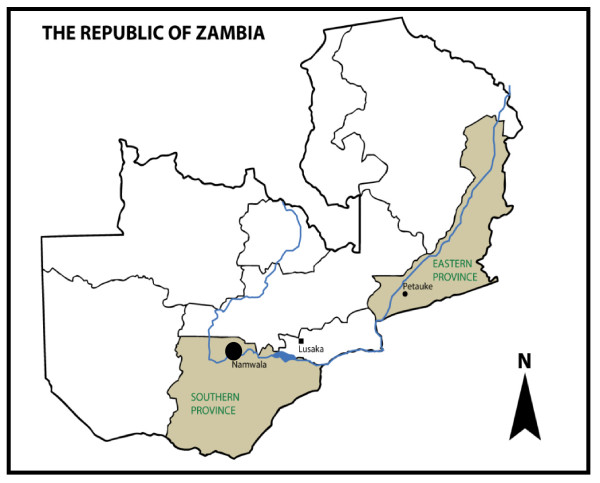
**Map of Zambia, showing the two provinces of Zambia that had reports of plague**. The study area, Namwala district in Southern province is highlighted.

The study was conducted in the southern Province of Zambia (Figure 1) in Namwala district (15°54'-15°56'S, 026°51'-026°54'E) which has had sporadic outbreaks of plague since 1997, after a major outbreak [5]. The area is located in a flood plain, where water levels are influenced by the yearly seasonal rains commencing from November to March. The climate is semiarid with a vegetation type of homogeneous short grass cover and some portions of the Savanna woodland. Sampling was done at 5 sites where clinical cases of plague have been reported. Fleas were collected from rodents trapped in the peridomestic areas (about 100 metres away from human dwellings) from June 2010-August 2011 by using the Sherman Trap (H.P. Sherman traps, Tallahassee, FL, USA). The fleas were collected from the captured rodents using an animal grooming comb after anaesthetising them with isoflurane. The fleas were brushed on a white cloth for visibility and easy collection. The collected fleas were stored in absolute ethanol before analysis after which they were identified as previously described [17,18]. The fleas were placed in pools of one-10 individuals (corresponding to the same animal host and flea species) and then tested for the presence of *Y. pestis *DNA. The rodents were identified to genus level following the field guide to mammals of Southern Africa [19].

For further analysis, the ethanol preserved fleas were rinsed with distilled water and subsequently dried on sterile filter paper in a laminar biosafety hood. The fleas were placed in the Eppendorf tubes with 100 μl of brain-heart infusion broth (Oxoid, Hampshire, England) and then triturated with a sterile pipette as previously described [1,15]. The triturated samples were then boiled at 95°C for 10 min and then followed by centrifugation for 10 sec at 10,000 *xg*, where 1 μl was used as a template for PCR testing. Negative control template employed brain-heart infusion broth only and fleas collected from a non endemic plague area. Briefly, PCR amplification was performed for the detection of the *Y. pestis *plasminogen activator gene using primers *Yp pla1 *(5'TGC TTT ATG ACG CAG AAA CAG G3') and *Yp pla2 *(5'CTG TAG CTG TCC AAC TGA AAC G3') as previously described [20]. The primers amplify a 344 bp region of the *Y. pestis *plasminogen gene. PCR was done using the Phusion™ flash high fidelity PCR master mix (Finnzymes Oy, Finland). The reactions were performed in a final volume of 10 μl containing 5 μl phusion flash PCR master mix, 0.5 μM of primer sets in 1 μl volume of each and 2 μl of PCR water. The Piko™ thermal cycler (Finnzymes Instruments Oy, Finland) was programmed at 95°C for 10 sec for initial denaturation, followed by 35 cycles consisting of 95°C for 1 sec, 58°C for 5 sec and 72°C for 15 sec. Final extension was given 72°C for 1 min. Specific *Y. pestis *detection was identified by the presence of a specific 344 bp DNA band on 1.5% agarose gel, stained with ethidium bromide and evaluated under UV transilluminator. The estimation of the sizes of PCR products was done according to the migration pattern of a 100-bp DNA ladder.

## Results

**Figure 2:**
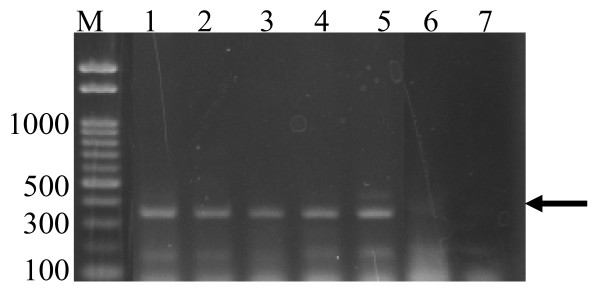
**Detection of *Y. pestis *plasminogen activator gene in fleas by PCR that were collected from rodents**. Lanes1 to 5, results of the positive fleas from the gerbils. Lane 6 is the negative control from fleas trapped in a non endemic area, while Lane 7 is the negative control of using brain-heart infusion broth as template.

PCR results for fleas collected from the rodents were as shown in Table 1. There were four species of rodents trapped. Of the 83 rodents, 79.5% were *Gerbillurus *species, 14.5% as *Rattus *species, 3.61% were *Mastomys *species and 2.41% as *Saccostomus *species. Thus, the gerbils formed the greater percentage of the rodents caught. They are also the ones that yielded a total of 219 fleas, which were harvested and subjected to PCR respectively. All the fleas observed were from the *Xenopsylla *species. Only 5 of the gerbils (7.58%; 95% CI = 2.82-17.5%) carried fleas that were positive for the *Y. pestis *plasminogen activator gene as shown in Figure 2 (indicated by an arrow). The expected PCR amplicon of 344 bp was amplified. Based on the total number of captured rodents with fleas, the percentage of rodents that had fleas with amplified DNA from *Y. pestis *translated to 6.02%.

**Table 1:** PCR results for fleas collected from various rodents (n = 83)

Rodent host	Number of Rodents sampled	Rodents found with plague positive fleas	Percentage
*Gerbillurus*	66	5	7.58 (95% CI = 2.82-17.5%)

*Rattus*	12	0	0

*Mastomys*	3	0	0

*Saccostomus*	2	0	0

**Total**	**83**	**5**	**6.02** **(95% CI = 2.24-14.1%)**

## Discussion

Rural Africa reports more than 90% of all plague cases worldwide [21]. The cases occur in areas where the disease is endemic. In these areas, facilities for the surveillance and diagnosis of plague are absent. In this study the focus was made on the potential vector and efforts were made to detect the causative agent of plague, using PCR targeting the plasmid encoded plasminogen activator gene. The PCR assay targeting this gene has been employed by a number of workers in similar studies [1,15,16,22]. The plasminogen activator gene is unique to and highly conserved in *Y. pestis *[1]. In our study fleas were targeted as they are easier and safer to handle than mammalian tissue [1,16]. PCR was used rather than bacterial isolation because it is safer; most African countries, reporting this disease may not have appropriate bio-containment facilities to handle this dangerous pathogen. Furthermore the PCR method has advantages over other methods as it is simple to perform and produces results within the shortest possible time. In this study, *Y. pestis *was detected from 6.02% of the rodents harbouring fleas. From these rodents, the gerbil was the only rodent observed to have had fleas with evidence of plague. This suggests the involvement of gerbils as hosts of fleas carrying *Y. pestis *as reported in Asia [23-25]. Gerbils tend to live in family groups that inhabit and defend discrete, permanent burrow systems. In the burrows fleas which cannot survive outside will live on the host and have limited access to other rodents in neighbouring systems. In our study area, this reasoning could apply as to why outbreaks occur in summer. In Zambia the plague season is usually during the rain season (November to March) when rodents leave their flooded burrows to nearby human dwellings where they seek refuge with their fleas. Observations on the rodent and flea populations in our study area indicate that, there is an abundance of these animals during the rain season [5].

The use of PCR to rapidly identify *Y. pestis *infected fleas is important in assessing human plague risks [1,15]. Detection of plague infected fleas in an area, can lead to intervention strategies, such as residual spraying with insecticides. This would help break the transmission of plague, as it occurs primarily via the bites of infected fleas [8].

## Conclusion

We conclude that the PCR assay for the detection of the plasminogen activator gene of *Y. pestis *DNA may be important in the monitoring and detection of plague foci areas and reservoirs in southern Africa.

## Abbreviations

PCR: Polymerase chain reaction; Yp pla: *Yersinia *pestis plasminogen activator.

## Competing interests

The authors declare that they have no competing interests.

## Authors' contributions

HBM, SKL and KD undertook sample collection and laboratory experiments. NI, SH and SC contributed to the design of field data collection. MAS, KBS and WB contributed to the design and writing of the manuscript. All authors read and approved the final manuscript.
